# Determinants of selective reporting: A taxonomy based on content analysis of a random selection of the literature

**DOI:** 10.1371/journal.pone.0188247

**Published:** 2018-02-05

**Authors:** Jenny T. van der Steen, Cornelis A. van den Bogert, Mirjam C. van Soest-Poortvliet, Soulmaz Fazeli Farsani, René H. J. Otten, Gerben ter Riet, Lex M. Bouter

**Affiliations:** 1 Department of Public Health and Primary Care (PHEG), Leiden University Medical Center (LUMC), Leiden, the Netherlands; 2 Department of Primary and Community Care, Radboud university medical center, Nijmegen, the Netherlands; 3 Division of Pharmacoepidemiology & Clinical Pharmacology, Utrecht University, Utrecht, the Netherlands; 4 Program on Aging, Netherlands Institute of Mental Health and Addiction (Trimbos-instituut), Utrecht, the Netherlands; 5 Division of Pharmacoepidemiology & Clinical Pharmacology, Utrecht University, Utrecht, the Netherlands; 6 Medical Library, Vrije Universiteit, Amsterdam, the Netherlands; 7 Department of General Practice, University of Amsterdam, Academic Medical Center, Amsterdam, the Netherlands; 8 Department of Epidemiology & Biostatistics, VU University Medical Center, Amsterdam, the Netherlands; 9 Vrije Universiteit, Faculty of Humanities, Department of Philosophy, Amsterdam, the Netherlands; Universite Paris Descartes, FRANCE

## Abstract

**Background:**

Selective reporting is wasteful, leads to bias in the published record and harms the credibility of science. Studies on potential determinants of selective reporting currently lack a shared taxonomy and a causal framework.

**Objective:**

To develop a taxonomy of determinants of selective reporting in science.

**Design:**

Inductive qualitative content analysis of a random selection of the pertinent literature including empirical research and theoretical reflections.

**Methods:**

Using search terms for bias and selection combined with terms for reporting and publication, we systematically searched the PubMed, Embase, PsycINFO and Web of Science databases up to January 8, 2015. Of the 918 articles identified, we screened a 25 percent random selection. From eligible articles, we extracted phrases that mentioned putative or possible determinants of selective reporting, which we used to create meaningful categories. We stopped when no new categories emerged in the most recently analyzed articles (saturation).

**Results:**

Saturation was reached after analyzing 64 articles. We identified 497 putative determinants, of which 145 (29%) were supported by empirical findings. The determinants represented 12 categories (leaving 3% unspecified): focus on preferred findings (36%), poor or overly flexible research design (22%), high-risk area and its development (8%), dependence upon sponsors (8%), prejudice (7%), lack of resources including time (3%), doubts about reporting being worth the effort (3%), limitations in reporting and editorial practices (3%), academic publication system hurdles (3%), unfavorable geographical and regulatory environment (2%), relationship and collaboration issues (2%), and potential harm (0.4%).

**Conclusions:**

We designed a taxonomy of putative determinants of selective reporting consisting of 12 categories. The taxonomy may help develop theory about causes of selection bias and guide policies to prevent selective reporting.

## Introduction

Complete, accurate and timely reporting of all (study protocol-stipulated) outcomes is essential for syntheses of research to be valid and as precise as possible [1, 2]. Complete or unselective reporting refers to both unselective publication of all results of a study as well as unselective or complete reporting within publications on all planned outcomes [3]. In other words, all planned outcomes should be reported on within a reasonable time frame (and the exploratory nature of analyses with any unplanned outcomes should be disclosed).

Selective reporting leads to bias if specific results remain unpublished because the decision to report depends on the nature of the results (e.g., direction or magnitude of the target association). Reporting bias is an important threat to the validity of systematic reviews which clinicians, researchers, policy makers and citizens rely on. Reporting bias is wasteful, distorts the aggregate body of scientific evidence, threatens the credibility of science, but it may also result in suboptimal treatment or even in avoidable harm to, e.g., patients’ health. Therefore, in addition to validity and efficiency reasons, there is an ethical imperative to report all results including those of clinical trials [4, 5].

Journals increasingly require that protocols have been registered before commencing or completing a study to facilitate the detection of selective reporting and increase transparency, and there is some evidence that registration of trials is effective [6, 7]. However, around half of planned outcomes of clinical trials are not reported, and a third to half of registered clinical trials remain unpublished [8–11]. There are numerous reports that suggest that selective publication is a major problem in the clinical domain, but it is also pervasive in basic and translational research [12, 13] and in the social sciences [14]. Selective reporting occurs in various other types of studies and across various designs, e.g., trials with psycho-educational interventions, and quantitative observational and qualitative studies [11, 15, 16].

So-called “protocol-to-publication” and similar studies point to selective reporting of statistically significant results [11, 17, 18]. More generally, “preferred findings” are more often reported. Preferred findings are often statistically significant findings. However, there are exceptions such as in case of studies into adverse effects, or equivalence trials where no difference is preferred. As a consequence, findings preferred by key stakeholders in the research project at issue are likely overrepresented in the scientific literature [19].

Research has suggested that financial conflicts of interest may cause selective reporting (e.g., when studies are sponsored by industry [9, 11, 20, 21]), but non-financial conflicts of interest probably play an important role too. Causes of publication bias, and of reporting bias more generally, may relate to decisions taken by researchers and sponsors, and also decisions by editors [8, 9, 22, 23]. Some have argued that it is human nature to search for positive messages, which suggests that basically, all scientists are at risk [2, 24]. However, certain persons or environments may be at increased risk of selective reporting, such as junior researchers [25] and scientists in more competitive academic environments [26].

Despite numerous studies on selective reporting, there is no accepted taxonomy of its determinants and no explicit causal framework. A recently developed framework of non-publication [22] attempted to answer the questions “what?” (defining dissemination), “who?”(is to blame-actor/stakeholder) and “why?” (stakeholders’ motivations). However, it does not provide a single taxonomy of determinants and its scope was limited to clinical trials.

We aimed to develop a taxonomy of putative determinants of selective reporting. We therefore addressed the questions of “what are possible determinants of selective reporting?,” and “how are putative determinants of selective reporting best grouped based on its content?“.

## Methods

### Design, protocol and reporting

To develop a taxonomy of putative determinants of selective reporting, we combined principles of systematic reviews [27] with those of inductive qualitative content analysis [28, 29]. Before analyzing full-texts, we piloted search strategies, we piloted abstract and full-text eligibility criteria and reviewed procedures as detailed in the protocol (S1 File). We developed the study protocol based on the preferred reporting items for systematic review and meta-analysis *protocols* (PRISMA-*P*) guidelines [30,31] as far as applicable. In this article, we report applicable items from the PRISMA [32] guidelines for systematic review and applicable items from guidelines for the reporting of qualitative studies [33, 34].

### Eligibility criteria

We included articles that examined or suggested determinants of selective reporting. We searched for articles from any academic discipline reporting on studies employing any type of design based on empirical data, such as intervention studies (with any type of comparator), and observational studies. Additionally, to cover hypotheses on what drives selective reporting, we included non-empirical articles such as editorials presenting opinion, theoretical considerations and anecdotal evidence. To minimize duplication of determinants extracted from articles that were also selected for inclusion in reviews, we excluded reviews. The outcome, selective reporting, comprised non-publication and selective reporting within publications. Our review also covered the possible consequences of selective reporting, including publication bias and other types of reporting bias.

### Information sources

We searched PubMed, Embase, PsycINFO and Web of Science to cover a wide range of academic disciplines from inception to January 8^th^ 2015. We limited the search to the English, French, German and Dutch languages.

### Search strategy

The search strategy focused on selective reporting to avoid limitation to any preconceived determinants. We used terms for bias and selection combined with terms for reporting and publication (S1 File, Box 1), and we pilot tested the search strategy.

**Fig 1:**
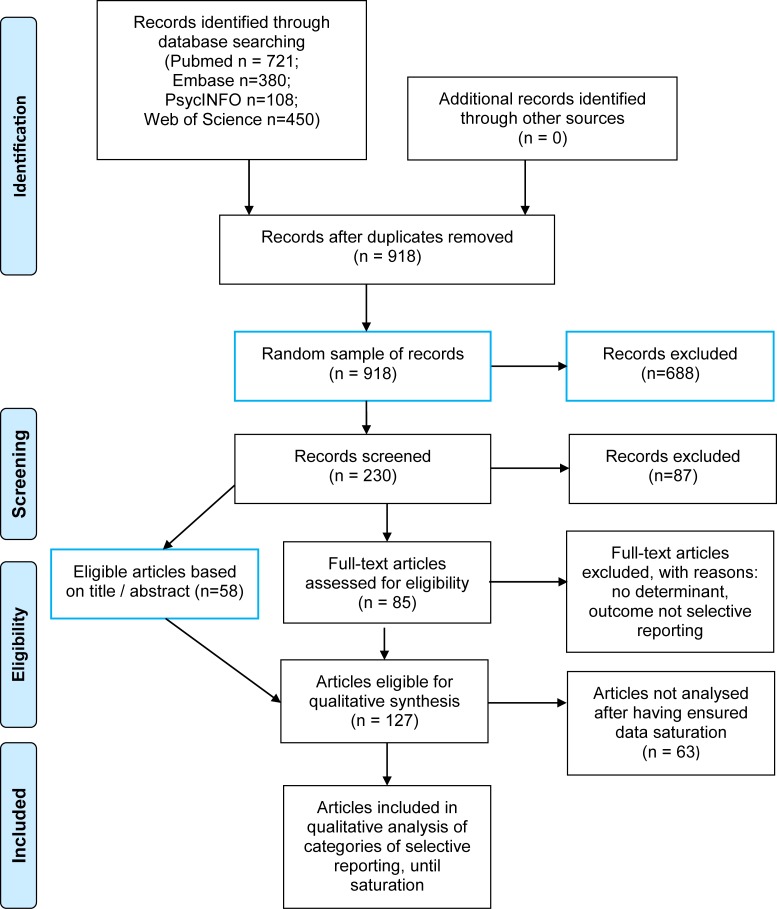
PRISMA flow diagram of identified and analyzed articles.

For reasons of feasibility of identifying determinants with qualitative content analysis (S1 File, Box 1), we retained a 25% random sample of the total of 918 hits after deduplication, from all databases searched from inception to January 8th 2015 (Fig 1). We used SPSS version 22’s random allocator function “random sample of cases” to randomly select a quarter comprising 230 hits.

### Selection of articles

Titles and abstracts were screened independently against the inclusion criteria by pairs of two reviewers (JTvdS, CAvdB and MCvS-P). If there was no abstract, we reviewed keywords. We used the first ten percent of abstracts (23) to test a shared understanding of the inclusion criteria and we discussed discrepant interpretations. Of the other abstracts, we calculated inter-rater agreement (percentage) of decisions on retrieval as full-text. We evaluated for eligibility all full-text articles and we calculated inter-rater agreement also for this step.

### Data collection process

Full-text data extractions were performed by pairs of researchers (JTvdS, CAvdB, MCvS-P, SFF, and GtR). JTvdS, CAvdB and MCvS-P piloted a standardized full-text data extraction spreadsheet using three empirical and non-empirical articles not included in the random sample. Initially, the reviewers extracted data independently, but after, for each pair of analysists, the analysis of a few articles made it clear that determinants were identified consistently, one reviewer extracted data with verification by another (S1 File, paragraph Data collection process).

### Data items

At the level of the article, we abstracted year of publication, academic discipline and study design (S1 File, box 2). More than one determinant per article was possible. We assessed whether evidence of association of a determinant with the outcome was empirical, indicated an actor (stakeholder), referred to a cause or could possibly be interpreted as a cause (we thus explicitly assessed the degree of interpretation [35]). We assessed the type and scope of selective reporting (whether limited to a single medium, e.g., a specific journal) and the nature of the association between a putative determinant and selective reporting (if there was any association, the direction, and strength of association, if applicable).

### Analyses

The sample of articles and determinants was summarized using percentages. We used qualitative iterative and inductive content analysis to group determinants by content and form categories of determinants [29]. In particular, the reviewers (pair) extracted putative determinants in the context of the article, and the team subsequently discussed interpretations and categorization. In the content analysis, we coded putative determinants and subsumed them under meaningful categories without imposing any prior model. We considered determinants which were tested even when authors found them unrelated to selective reporting in the particular study, because different studies often vary in finding associations with outcomes. A single dataset with putative determinants was created after discussions on differences of interpretation.

Next, two researchers (JTvdS and CAvdB) independently categorized all determinants into higher-level groupings. This was a non-linear, iterative process as we classified batches of about 50 determinants followed by discussing all classifications of each batch before moving on to the next batch. We avoided overlap in categories [35] by adding descriptions (to serve as definitions) that we developed from the iterative classification of content. In reaching consensus about the categorization we often went back to previous classification work to adapt categories, or to the full-texts to ensure we understood the context. Thus, we constructed a structured list of categories of more specific determinants. An initial classification and any unresolved issues about further classifications were discussed with a third researcher (GtR) to achieve full consensus. Following the principles of content analyses [9], we counted the number of determinants per category for descriptive purposes of which categories were more and which were less prominent in the literature.

#### Saturation

We analyzed content of articles randomly selected from different decades (< 1980, 1980s, 1990s, 2000–2009, 2010–2015). We concluded analyses when saturation was reached. Saturated data ensure replicability in categories derived from content analyses, which in turn verifies and ensures comprehensiveness [35]. Saturation of categories was determined in two ways. First, during the process of analyzing the batches of determinants we assessed saturation prospectively, during the content analyses, in the usual way for qualitative analyses (i.e., no new categories emerged in the last analyzed articles). After having analyzed about 50 articles, we deemed the newly classified determinants not to be essentially different from those already classified (we could fit them in the categories we had developed), and we assumed saturation. This was confirmed after having analyzed one more batch of determinants representing 10% of articles (13 of 127). Second, retrospectively, after having analyzed the articles, we verified quantitatively how many articles had been analyzed when the first determinant of each category emerged.

#### Subgroup analyses

We assessed the extent to which various determinants were based on empirical studies or (solely) on opinion (as planned and described in the protocol, S1 File). Further, to avoid inclusion of categories exclusively comprising determinants that were consistently unrelated to outcome, we calculated the proportion of articles that (quantitatively or qualitatively) reported a non-significant or no association, or an unexpected direction of an association between a determinant and selective reporting.

## Results

### Selection of articles for analyses

We identified 918 unique records, and we included 64 records (articles) in the final analysis (Fig 1). The inter-rater agreement between independent assessors on the need to retrieve the full-text as assessed for 90% of the records was 72% (150/207; agreement about 143 records, doubt about 7, no agreement about 57). The initial agreement about eligibility of full-text assessment was 78% (66/85). All initial disagreements were resolved through discussion.

### Description of the dataset: Articles and determinants

Of the 64 articles analyzed, 48 (75%) concerned clinical medicine, and 51 (80%) were published in 2000 or later (Table 1). Almost half of the articles (31) were non-empirical. The empirical studies were mostly observational and quantitative; we found one RCT [36].

**Table 1:** Characteristics of the 64 analyzed articles.

Characteristic	%	n
Year of publication		
< 1980 1980s 1990s 2000–2009 2010–2015	2 3 16 44 36	1 2 10 28 23
Academic discipline		
Clinical medicine Biomedicine / Life sciences Humanities No specific discipline	75 9 11 5	48 6 7 3
Type of study / study design		
Non-empirical (reflective / theoretical) Observational quantitative, longitudinal* Observational quantitative, cross-sectional* Case description / anecdotal Simulation / modelling^†^ Randomized trial^‡^ Qualitative Mixed methods (integrated) Review of reviews	48 27 9 5 5 2 2 2 2	31 17 6 3 3 1 1 1 1

* Observational quantitative studies included: comparisons of publications (n = 5), comparison of registry records with publications (4), of protocols with publications (4), of submitted with accepted papers (4), of abstracts with publications (4), of public funder database with publications (1) and of industry database with Medline records (1)

^†^ Mathematical simulations of reporting bias, subjective decision-making in peer-review, and the selection process in publication bias, whether purely hypothetical or with use of empirical data

^‡^ The RCT assessed the effect of blinded peer-review on reviewers’ and editors’ decisions about manuscript acceptance [36]. The determinant was prejudice in the peer-review process, and the outcome was non-publication, considering that the editor’s decision dictates whether the manuscript is published

We extracted 497 determinants from the 64 articles (median 6; range 1 (9 articles) to 22 (3 articles; Table 2; S2 File). Twenty-nine percent (145 determinants) concerned empirical evidence of associations with selective reporting. If an actor (stakeholder) was mentioned (41%, 204 determinants), it was the investigator in about half of cases (110 determinants). In 79% of cases, an association with selective reporting was found or postulated (all in the hypothesized or expected direction).

**Table 2:** Characteristics of the determinants (n = 497), outcomes and their associations.

Characteristic	% (n)
Evidence of association of determinant with outcome	
Empirical Non-empirical (e.g., from viewpoint, or opinion in discussion section, or inference from the literature or theoretical study)	29 (145) 71 (352)
Actor (stakeholder)	
involved *investigators or authors* *editors or journals* *reviewers* *sponsors or industry* *government* *analyst* no actor mentioned	41 (204) *22 (110)* *11 (57)* *4 (18)* *3 (17)* *0*.*2 (1)* *0*.*2 (1)* 59 (293)
Interpretation of association (hypothesized, whether confirmed or not) in terms of possible causal pathways*^†^	
Describes a cause Allows for a single and clear interpretation of cause Unclear cause or multiple causal interpretations are possible	22 (111) 20 (98) 58 (288)
Type of selective reporting outcome	
Non-publication Selective publication in general Selective reporting within publication Reporting bias Other (delayed publication‒which risks e.g., no uptake in reviews)	59 (292) 16 (78) 14 (70) 11 (53) 1 (4)
Scope of selective reporting outcome	
Within a single medium (journal or conference) General	13 (66) 87 (431)
Reported association between determinant and outcome^†^	
Present (confirmed) No association	79 (393) 21 (104)

Note: the table is based on the pre-planned items for description of 497 determinants abstracted from the 64 articles

* Examples: “The company often owns the study database and controls decisions about publication and release of data” (describes a cause); authors reported “lack of time” as a reason (allows for a single and clear interpretation of cause‒ a cause is implicated (lack of time), but that cause itself begs a more detailed explanation (how does the reporting compete with other duties and why?)); sample size (unclear cause or multiple causal interpretations are possible‒ such as with larger sample size more power, more collaborators, more rigorous design, more quality checks etc.)

^†^Note that a possible causal interpretation of a determinant under study (a hypothesis) does not necessarily mean that in each case a causal association with the outcome was actually confirmed in the particular study or in the narrative (finding no association was still possible as an empirical result, or a possible causal association could be denied in a comment)

### Categories of determinants

Table 3 lists the 12 categories that emerged from coding the extracted determinants, along with descriptions and examples. *Focus on preferred findings* was the largest category (180 determinants, 36%), which included, for example, significance chasing. This concerned empirical data in 17% (30/180) of cases, and in 93% (168/180) of cases the original authors postulated that a focus on preferred findings was positively associated with selective reporting. By contrast, the second largest category (109 determinants, 22%), *poor or flexible research design*, was based on empirical findings in half of the cases, and the original authors mentioned a positive association in 57% of cases. The other 10 categories occurred less frequently (8% or less) yet represented distinct concepts. References to the 64 analyzed articles are provided per category, as a supplement (S3 File).

**Table 3:** Taxonomy of determinants (n = 497) resulting from the inductive qualitative content analyses.

Determinant classification, category	Description	Examples	% (n in full sample)	% empirical result* (n in category /-per row)	% any relationship^†^ (n in category /per row)
*1*. *Focus on preferred findings*	A focus on finding results that match preferences, mostly statistically significant or otherwise positive findings, wishful thinking and acting	Significance chasing, finding significant results, larger effect size, suppressing publication of unfavorable results, not being intrigued by null findings	36 (180/497)	17 (30/180)	93 (168/180)
*2*. *Poor or flexible research design*	Attributes of study design relating to power and level of evidence provide much leeway in how studies are performed and in interpretation of their results	Not a controlled or blinded study, study protocol unavailable, small sample size	22 (109/497)	50 (54/109)	57 (62/109)
*3*. *High-risk area and its development*	Area of research or discipline or specialty including its historical development and competitiveness, the currently dominant paradigms and designs, and career opportunities	Ideological biases in a research field, area with much epidemiological research versus clinical or laboratory research (“hard sciences”), humanities, experimental analytic methods, “hot” fields, publication pressure in the specific field	8 (39/497)	31 (12/39)	72 (28/39)
*4*. *Dependence upon sponsors*	Financial conflict of interest resulting in lack of academic freedom	Requirements and influence of funding source with financial interests in study results	8 (38/497)	34 (13/38)	82 (31/38)
*5*. *Prejudice*	A conscious or unconscious belief that may be unfounded, and of which one may or may not be aware	Prior belief about efficacy of treatment, author reputation or gender bias in the phase of review	7 (33/497)	24 (8/33)	82 (27/33)
*6*. *Lack of resources*, *including time*	Insufficient manpower or finances	Lack of time resulting from excessive workload, or lack of personnel due to life events	3 (17/497)	18 (3/17)	100 (17/17)
*7*. *Doubts about reporting being worth the effort*	Weighing investment of time and means versus likelihood of gain through publication	Anticipating disappointment of yet another rejection or low chances of acceptance of a manuscript, belief that findings are not worth the trouble	3 (16/497)	6 (1/16)	100 (16/16)
*8*. *Limitations in reporting and editorial practices*	Constraints and barriers to the practice of reporting relevant detail	Journal space restrictions, author writing skills	3 (14/497)	71 (10/14)	50 (7/14)
*9*. *Academic publication system hurdles*	Various hurdles to full reporting related to submission and processing of manuscripts (other than reporting) including those that represent an intellectual conflict of interest	Solicited manuscripts, authors indicating non-preferred reviewers, editor’s rejection rate	3 (14/497)	36 (5/14)	57 (8/14)
*10*. *Unfavorable geographical or regulatory environment*^*‡*^	Geographical or regulatory environment that affects how research is being performed	Continents under study included North America, Europe and Asia; few international collaborations; no governmental regulation of commercially sponsored research	2 (12/497)	67 (8/12)	75 (9/12)
*11*. *Relationship and collaboration issues*	Intellectual conflict of interest between reporting and maintaining good relationships	Disagreements among co-authors and between authors and sponsors, sponsors prefer to work with investigators who share the sponsor’s position	2 (8/497)	13 (1/8)	100 (8/8)
*12*. *Potential harm*	Publishing data can harm individuals	Risk of bioterrorism, or confidentiality restriction	0.4 (2/497)	0 (0/2)	100 (2/2)
(13) Not specified	Referring to a stakeholder only	Selective publication not caused by editors	3 (15/497)	0 (0/15)	67 (10/15)

*Empirical result as described in Table 2, first row

^†^Any relationship, and in the expected direction if any relationship was being hypothesized, versus no relationship. None of the hypothesized relationships in empirical result were found to be in the opposite direction

^‡^We aimed to consistently include a direction in all category names. However, the work from which we abstracted the determinants for this category was probably less theory driven (often not providing background or a hypothesis of direction), and more data driven (combining countries in order to attain sufficiently large groups). For example, manuscripts from the US versus all other countries was tested and there were very few manuscripts from other countries. This made it difficult to find a term that clearly describes direction. We therefore used “unfavorable” without further specification

### Saturation

**Fig 2:**
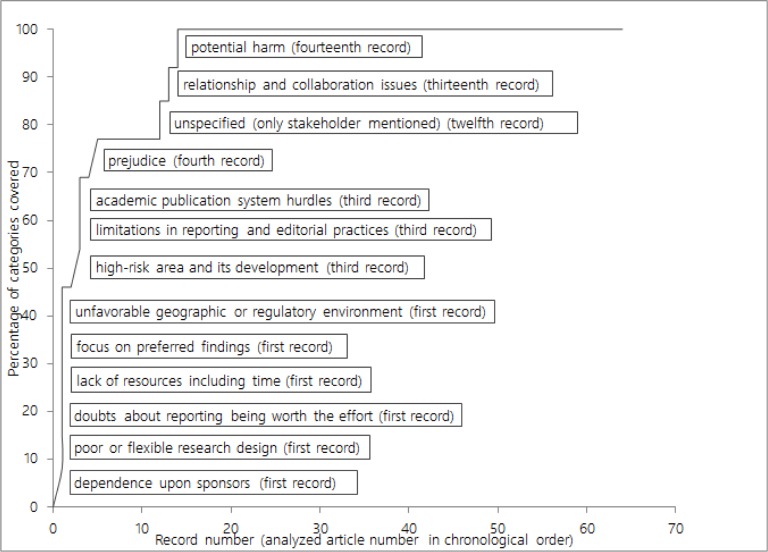
Saturation graph. The horizontal axis displays the articles (records) in chronological order of analysis. The vertical axis displays the percentage of the 12 (plus 1 unspecified) determinant categories containing at least one determinant. The labels describe which determinant category appeared for the first time in which record.

The first determinant in each category usually emerged after analysis of only a few articles (Fig 2). After having extracted 120 determinants (of 497, 24%) from 14 articles, all categories comprised one or more determinants. Assuming that a category emerges upon identification of at least one or more determinants that are dissimilar from other categories, this indicates that saturation could have been reached earlier, with fewer than the set analyzed articles.

### Relationships and similarities between categories in view of possible causality

The descriptions clarified boundaries between categories that were conceptually close, such as *high-risk area and its development*, and *unfavorable geographical and regulatory environment*. Both categories represented a wider environment than the research team or institution.

The categories of *unfavorable geographical and regulatory environment* and *academic publication system hurdles* were distinct as these included determinants that did not clearly refer to a possible hypothesis regarding a mechanism or cause of selective reporting (an example is provided as a Footnote to Table 3). By including these categories, we were able to classify all determinants, except for 15 that only mentioned a stakeholder (actor) as the source of selective reporting (or the denied source, such as when an actor is believed not to cause selective reporting, in which case we recorded no association between determinant and the outcome of selective reporting).

We found six described instances of interaction between determinants (effect modification). We counted these as classified with the main determinant only. The interactions all clearly described causes. For example, “Outcomes could be deemed post hoc to have little clinical relevance if they fail to show significant findings and may thus be omitted when accommodating space limitations” [37]. In this case, the interaction between *focus on preferred findings* and *limitations in reporting and editorial practices* was classified under the first. By contrast, we classified another interaction, between a *focus on preferred findings* and *high-risk area and its development* under the latter as the primary category: *“*Early in the history of a research domain results in either direction are important news but [that] later, when the preponderance of evidence has supported one direction, significant reversals are often more important news than further replications.” [38].

## Discussion

We developed a taxonomy of putative determinants of selective reporting in science based on saturated qualitative analyses of a representative sample of the relevant literature. The taxonomy clusters determinants in a meaningful way. It consists of 12 mutually exclusive categories along with descriptions and examples to clarify boundaries and differences between the categories. The taxonomy should give structure and depth to commonly used expressions such as significance chasing (placed in the category *focus on preferred findings*) and conflict of interest (financial: *dependence upon sponsors* and *relationship or collaboration issues*; intellectual: *relationship or collaboration issues* and *academic publication system hurdles*).

Two categories, *focus on preferred findings* and *poor or flexible research design*, covered over half of the determinants we found. These related mostly to choices of individual researchers or teams. The individual or team level was also referred to in six of the 10 other categories (*prejudice*, *dependence upon sponsors*, *lack of resources including time*, *doubts about reporting being worth the effort*, *limitations in reporting and editorial practices*, which refers to individual editors and authors, and *relationship and collaboration issues*). Four categories referred to the wider environment. These were the categories of *academic publication system hurdles* and *potential harm*; the other two included determinants that often lack clear direction or hypothesis (e.g., when a range of disciplines or countries are compared based on distributions): *high-risk area and its development*, and *unfavorable geographic or regulatory environment*.

### Related conceptual work on selective reporting

The basis of the framework of Bassler et al. [22] comprised 50 highly cited articles published until 2012 identified in Web of Science, and consensus among 10 experts. Our work represents a wider scope of literature. For example, potential harm through bioterrorism was identified through veterinary medicine literature [39]. Also, Web of Science identified less than half of the articles in our sample. We included expert views and aggregate understanding, and determinants that may not have been studied well, yet in a different manner: through analyzing editorials, comments, and the full articles including introduction and discussion sections. We used explicit and transparent inductive qualitative research methods to cover the broad range of putative determinants in the literature. In contrast, Bassler et al. [22] focused on actors and motivations, which complements our work to help understand the multi-causality and multiple system pressures on and rewards for individuals and teams.

### Limitations and strengths

The combined quantitative and qualitative approaches including two different ways ‒prospectively and retrospectively‒ to check saturation increased the likelihood of having captured all relevant categories of determinants and served as an internal validation of our approach. However, the data did not suffice to discern patterns of determinants by academic field or strength of evidence. Our work does not cover all possible single determinants. We do believe that the framework with categories likely captures the categories that fit with the large majority of determinants. For example, we did not find lack of a statistician on the team being a putative determinant, but depending on the hypothesis of how it may relate to selective reporting, it may fit, for example, with *poor or flexible research design* or *focus on preferred findings*, or *lack of resources including time*. Further, in our work, discussion to reach consensus was essential because abstracting possible determinants without a prior framework was not a straightforward endeavor, as evidenced by modest initial agreement (72% and 78%) about inclusion of articles in reviewing abstracts and full-text.

Not publishing research outcomes is unethical. Our findings, however, raise questions about possible rare but legitimate reasons to report selectively or to not publish research. Obviously, potential harm can be considered a legitimate reason, when publication may involve misuse by e.g., terrorists, or involves breaking confidentiality restrictions. Fatally flawed research probably should also not be published. However, poor design is preferably prevented in the first place, the academic reviewing system is in place to improve quality and to prevent fatally flawed work to be published or to be included in reviews and meta-analyses [40]. Future guidance may clarify what should be published in such cases.

### Future studies

New research, using various methods, should verify the categories we created. The categories and their interrelations may clarify causal pathways and inform theory. (Complex) interventions should probably and most fruitfully address several determinant categories. So far, most empirical work has been performed on poor or flexible research design, but not all findings refer to clear causes and therefore cannot be used to formulate interventions (such as studies examining the association of sample size with selective reporting of positive findings). Future research should also employ qualitative methods to address researchers’ daily decision making and balancing of interests to better understand causal mechanisms and the multiple factors involved.

The taxonomy may also help plan studies on risk profiling (e.g., research domains in which flexible designs are commonly used, or where a particular mission prevails) which in turn may inform efficient policy development on responsible conduct of research. We suggest our work to promote a constructive debate on causes of reporting bias. It is hoped that it will contribute to decrease the mostly deleterious phenomenon of selective reporting in modern science.

## Supporting information

S1 PRISMA Checklist (DOC)

S1 File Study protocol dx.doi.org/10.17504/protocols.io.jz3cp8n. (DOCX)

S2 File Dataset with determinants. (XLS)

S3 File References to the 64 articles included in the determinant analysis, per category. (DOCX)
