# Supplementary material for: Determinants of selective reporting: A taxonomy based on content analysis of a random selection of the literature
Source: PLoS One. 2018 Feb 5;13(2):e0188247. doi: 10.1371/journal.pone.0188247 (PMC5798766; doi:10.1371/journal.pone.0188247)
Supplement: S1 File — (DOCX) [file pone.0188247.s002.docx]

Supplemental file

**S1 . Study protocol**

Determinants of selective reporting: protocol for a review and content analysis of a random selection of the literature

Authors: Jenny T. van der Steen, PhD,* Mirjam C. van Soest-Poortvliet, MSc, Cornelis A. van den Bogert, MSc, René H.J. Otten, MSc, Gerben ter Riet, MD, PhD, and Prof. Lex M. Bouter, PhD

*Corresponding author. Department of Public Health and Primary Care (PHEG), Leiden University Medical Center (LUMC), Hippocratespad 21, 2300 RC Leiden, the Netherlands (primary affiliation); and Department of Primary and Community Care, Radboud university medical center, Nijmegen, the Netherlands; Email address: [jtvandersteen@lumc.nl](mailto:jtvandersteen@lumc.nl)

ABSTRACT

**Background:** Selective reporting distorts the aggregate body of scientific evidence, wastes resources and can harm patients’ health and the credibility of science. Selective reporting may result from a focus on preferred findings by researchers and others stakeholders. However, it is unclear if some persons or environments are at greater risk of selective reporting than others. Our review assesses what is known and what is postulated about determinants of selective reporting in the scientific domain.

**Methods/design:** Using search terms for bias and selection combined with terms for reporting and publication, we systematically search the PubMed, Embase, PsycINFO and Web of Science databases. A 25 percent random selection of records is reviewed for inclusion by at least two reviewers based on title and abstract if available and, if needed, the full text. Inter-rater agreement is calculated. Examining the content of the entire article, including, for example the discussion section, we extract phrases mentioning determinants. From this, we compile a structured list of possible determinants. The results are also categorized by type of source (empirical result or view, study design, and academic discipline if variability suffices). We follow the PRISMA-P and PRISMA reporting guidelines for systematic reviews where applicable.

**Discussion:** We use principles of the systematic review along with principles of qualitative content analysis to focus on the nature of possible determinants of selective reporting. Our review includes both empirical findings and theoretical considerations. It will identify priority questions for further research on mechanisms of selective reporting. Furthermore, it will help in assessing risk of selective reporting. Such “risk profiling” may inform effective policy development on responsible conduct of research. The approach of combining quantitative and qualitative assessment techniques is expected to optimally inform well-targeted intervention studies and inspire policy development.

Subject: (Medical) Research Methods

Keywords: Research methods, Publication bias, Scientific misconduct, Qualitative research, Systematic review

**Background**

Unselective reporting of research findings is essential for research syntheses to be valid, especially because we do not fully understand this phenomenon. Therefore, there is an ethical imperative of reporting all results of, e.g., clinical trials [1]. However, around half of planned outcomes of clinical trials are not reported, and a third to half of registered clinical trials remain unpublished [2-5]. Selective reporting results in bias if reporting depends on the results, such as on the magnitude of the target association or on variables associated with it. This includes bias in the reporting of outcomes and other items within publications, and publication bias if manuscripts are not published at all. Selective reporting is not restricted to clinical trials but it is pertinent also to quantitative observational and qualitative studies [6,7]. Reporting bias is an important threat to the validity of systematic reviews which clinicians, researchers and policy makers rely on. Reporting bias therefore not only distorts the aggregate body of scientific evidence and threatens the credibility of science, but also results in waste of resources and may result in suboptimal treatment or even in avoidable harm to patients’ health.

So called “protocol-to-publication” and similar studies point to selective reporting of statistically significant results (e.g. [8-10]). More generally−also considering, for example, equivalence trials and adverse effects, where authors may prefer non-significance−there may be a focus on preferred findings resulting in its reporting being more likely than results that are considered to be less desirable. As a consequence, published articles include relatively many false-positive (or preferred) findings.

Research has suggested that financial conflicts of interest cause selective reporting (e.g. when studies are sponsored by industry [10-12]) but non-financial conflicts of interest play a role as well. Causes of publication bias, and of reporting bias more generally, are decisions taken by researchers, sponsors and editors [2,3,13]. Some have argued that it is the human nature to search for positive messages and that therefore all persons are at equal risk [14]. However, certain persons or environments may be at increased risk of selective reporting such as juniors not receiving adequate feedback [15] and scientists in more competitive academic environments [16].

We lack a comprehensive overview of the determinants of selective reporting. Such an overview is important to effectively promote proper reporting behaviour. “Risk profiling” may enable, for example, the efficient use of educational or other strategies to prevent selective reporting. An overview of determinants and the extent to which they have been studied can also inform further research on determinants and mechanisms of selective reporting.

**Objectives**

We assess, systematically where possible, the question of *what are the determinants of selective reporting*. In addition to the empirical literature, we include theoretical considerations and published expert opinions on what drives selective reporting. This allows for comparing of determinants supported by empirical evidence with determinants often referred to in, for example, editorials and other publications based on expert comments. Comparing what is known from the empirical literature and what is postulated about determinants of selective reporting, further inspires research on insufficiently studied determinants.

Referring to the participants, interventions, comparators, and outcomes (PICO) framework, our “study population” (P) is diverse and may include researchers, sponsors, editors and any other actors involved in the reporting of study results. The intervention (I) and comparator (C) is not narrowly defined. We not only include articles on intervention studies to identify determinants of the outcome (O) selective reporting, but also articles on observational studies. Moreover, there may or may not be a comparator. Of the PICO elements, in our review, the population and outcome are the most important. The population in fact represents the determinants we are looking for, and is therefore purposefully left undefined a priori.

We anticipate that we cannot analyse all possible publications that suggest or substantiate any determinant of selective reporting. Our review aims at providing a structured list of putative determinants of selective reporting, grouped by its content to inspire next steps in the development of preventive policies and future studies. The review may also be helpful to raise awareness, train researchers, and promote a constructive debate on selective reporting and contribute to decrease the deleterious phenomenon of reporting bias in modern science.

**Methods/design**

**Design**

The overall design combines principles of a systematic review with those of a qualitative content analysis. This protocol was developed based on the preferred reporting items for systematic review and meta-analysis protocols (PRISMA-P) guidelines [17,18] as far as applicable, and we will use PRISMA guidelines to report the results and any amendments to the protocol. Because of the methodological nature of the review and selective reporting not being a health-related outcome, it is not eligible for inclusion in the PROSPERO database.

**Eligibility criteria**

We select published articles from any academic discipline reporting on studies employing any type of design based on empirical data, such as intervention studies (with any type of comparators), and observational studies. Additionally, we include non-empirical articles such as commentaries, editorials and views studies presenting expert opinions, theoretical considerations and anecdotal evidence. We exclude letters and replies to letters because they often address a single specific issue. We exclude reviews of primary studies on determinants of selective reporting to avoid undue duplication of determinants, although we will note reviews and refer to it in the Discussion section of our report.

We include articles that examine or suggest possible determinants of selective reporting in the scientific domain. We include articles on selective reporting comprising non-publication and selective reporting within publications, and its consequences−any bias that stems from it, including publication bias and other types of reporting bias (Figure 1).

Based on pilot searches, we found that a focus on preferred, typically positive, findings is often mentioned as the main determinant of selective reporting. On the other hand, selective reporting is sometimes *defined* in terms of reports relatively often representing preferred findings. We consider the preference to be an intermediate factor between the more ‘upstream’ determinants and the outcome selective reporting (Figure 1 and see also under “Outcomes and prioritization” a paragraph with related contents [18]).

**Information sources**

We search the PubMed, Embase, PsycINFO and Web of Science databases to cover a wide range of academic disciplines from inception to January 8th 2015. We limit the search to the English, French, German and Dutch languages. We do not check references.

**Search strategy**

The search strategy focusses on terms for bias and selection combined with terms for reporting and publication (Box 1, numbers 1 to 5). It purposefully does not limit the search by requiring terms aimed at finding possible determinants directly because the mapping of a wide range of possible determinants is the goal of the review.

The development of the search strategy included pilot testing. In the fall of 2014, we explored PubMed searching with “Publication bias” all fields. This yielded >7500 publications. “Publication bias” as a MeSH term without major still resulted in > 2800 hits. To arrive at a smaller number of hits retaining the most relevant articles, we developed a strategy on the basis of a sample of 18 articles from our personal literature databases which included determinants of selective reporting [19-33]. We balanced the sample to include both empirical and non-empirical work.

We checked MeSH terms in PubMed and over half (10 of 18 articles) [10, 12, 13, 19-25] had “Publication bias” as a major MeSH term (number 1 in Box 1), including with subheadings (Publication Bias/statistics & numerical data*) [23,24], Publication Bias/trends*[25]. One was not indexed in PubMed [26] and the other 7 of 18 articles were indexed differently: with the MeSH term Publication bias but not indicated as major [27], without MeSH terms yet with keywords [28,29], MeSH terms nor keywords [30], or with other MeSH terms [31-33]. Regarding the last, the three articles included Ioannidis’ hallmark article from 2005 was not found with the MeSH term Publication Bias. It was identified by a combination of “Publishing” and “Bias(Epidemiology)” as major MeSH terms which we added to our strategy (Box 1, number 2). Some other of the 7 articles shared the MeSH term research design (>300.000 hits), but the other keywords differed widely (e.g. guidelines, diagnostic research, some more specific keywords). Therefore, inclusion of an additional wide range of MeSH terms for the strategy to also include these articles was not feasible. We did examine other search terms related to selective reporting, guided by the main terms in Figure 1. However, other terms were infrequently used or represented terms that covered a broader content, such as “dissemination bias” (3 hits in PubMed).

We assessed how useful the inclusion of different types of non-empirical articles was. We excluded articles less likely to express experts’ views or reflect upon a broad scientific basis of empirical work, such as biography and letters (Box 1, number 7). To examine if editorials were a useful addition, two researchers independently evaluated 40 editorials as full text (these often do not have an abstract), and after discussion, we included 20. We screened other types of articles and estimated that about one-third would be included. We developed similar search strategies in the other databases (Box 1).

Because of considerations of feasibility and efficiency of identifying determinants from all texts, we evaluate a 25% random sample of the total of 918 hits after de-duplication, from all databases searched from inception to January 8th 2015. That is, further testing of abstract reviewing showed that from many abstracts it was not very clear if they would include a determinant or not. For example, if the abstract reported strategies to address selective reporting, the full text article is expected to refer to causes or other determinants of selective reporting even if these were not reported in the abstract. In other cases, interpretation as a determinant was difficult without reading more about the context of a study. The content analysis includes reflection upon possible presence of determinants and subsequent discussions. These are very time consuming. For proper review, we therefore decided to reduce the sample, and based on the experience through developing the search strategy, 25% seemed feasible.

**Data management**

De-duplication of references is performed in Reference Manager, maintaining the reference from PubMed if available. Reference Manager generates the unique identifiers which are maintained in other software in which the data are managed and analysed. We use the SPSS random allocator function “random sample of cases” to randomly select a quarter comprising 230 hits.

Data abstracted from abstracts, and from full text are entered into two separate spreadsheets. After independent review, the spreadsheets with evaluations of two reviewers are combined to facilitate comparison and discussion, both for the abstract and for the full text spreadsheet. Response options are coded to enter numerical values whenever possible. Open-ended text is entered for factors that need description, and for notes and comments. The spreadsheets are imported into SPSS for counts needed for a PRISMA flow diagram and for other descriptive analyses such as crosstabs (e.g. main category of determinant by empirical versus non-empirical article).

**Selection process**

Titles and abstracts are screened independently against the inclusion criteria by pairs of two out of three reviewers involved in the title/abstract evaluation (JTS, MCSP and CAB). Two reviewers independently evaluate each abstract. If there is no abstract, we also review keywords. Ten percent of articles (23) is evaluated initially to test a shared understanding of the inclusion criteria, after which discrepancies are discussed. The abstracts are coded 0 if excluded, 1 if included, 2 if unsure and need to evaluate the full text to determine if the abstract is included. A value of 3 is assigned if the reviewer does not know how to code the abstract in case of which another reviewer evaluates the particular abstract. Full text of publications is retrieved if the researchers both assign code 1 or 2. Disagreements (if only one of the reviewers assign code 0) are resolved through discussion with the three reviewers.

The reviewers are not blinded for the article’s authors or institutions. We plan to calculate inter-rater agreement (percentage) of references that should be retrieved as full text (code 1 or 2 versus 0). We calculate inter-rater agreement on the next set of 90% of the abstracts.

**Data collection process**

Similarly, full text data extractions are performed in pairs, but at least four researchers (JTS, MCSP, CAB and GR) will be involved. We first evaluate for eligibility all full text articles assigned a 2 (unsure) in the previous abstract screening, and again calculate inter-rater agreement for this intermediate step. The standardized full text data extraction spreadsheet is pilot tested by three researchers (JTS, MCSP and CAB) using three articles not included in the random sample which cover empirical and non-empirical articles. We aim at independent data extractions, but once rules have been developed to identify determinants consistently, we may resort to data extraction of articles by one researcher with checking by the other.

No authors will be contacted in case of missing data. We examine included articles for possible overlapping reports or duplicates not identified earlier through processing the data, by searching Reference Manager for first and last author names.

**Data items**

Evaluating full text references, we abstract the items listed in Box 2. More determinants per article are possible. The items are collected at the level of the determinant, except for academic discipline, design, any preventive measures mentioned in the article and room for comments (Box 2, a, b, l, m) which are assessed at the article level.

**Outcomes and prioritization**

As also described under “Eligibility criteria” (and in Figure 1 and Box 2, item g) the outcome is selective reporting, which involves both non-publication and selective reporting within publications, and any reporting bias that stems from it. There is no prioritization of outcomes. Note that a focus on preferred, typically positive, findings may be an intermediate factor (visualised in Figure 1) as it is often mentioned as the main determinant of selective reporting but we might also find determinants of “significance chasing.”

**Risk of bias individual studies**

The reviewers are not blinded to the authors and results of the articles. Because the individual studies employ widely different designs (Box 2, item b), risk of bias tools such as the Cochrane tool for RCTs [34], and a tool to evaluate trial registration protocol-to-publication studies [35], apply to only a small subset of included studies. We are aware of appraisal criteria that apply to both quantitative and qualitative research [36], but these rate the quality of the reporting and they do not apply to opinion pieces without empirical data. We do, however, address the question of which type of studies are the sources of the determinants and if associations with selective reporting are in the same direction. Generalizability is stronger for determinants found in more studies or articles [37], studies from different academic disciplines (Box 2, item a), and those arising from more robust designs (order of items b and d in Box 2, except for mixed methods in b). For certain study designs, we collect additional information that refers to the quality of the study (items e, f, k in Box 2).

**Data synthesis**

We plan to report a list of the identified putative determinants of selective reporting presented in meaningful groupings. We do not anticipate a quantitative synthesis (meta-analysis) of the study data, such as on strength of quantitative associations because of the heterogeneity of the literature that includes possible determinants of selective reporting.

Essentially, our data synthesis forms a qualitative content analysis [38]. Researchers work in pairs to first independently interpret findings to abstract phrases that cover determinants from full article texts. Discussion to resolve any differences in interpretation results in a list of putative determinants. Two researchers categorize the determinants into higher-level groupings. Again after discussion, this should result in a structured list, a framework of groupings of more specific determinants. Following the principles of content analyses [38], we count the number of times we find a category of determinants or specific determinants, explicitly considering also determinants which were tested but found unrelated to selective reporting.

However, if more and comparable studies report a quantitative estimation of an association such as Odds Ratios or Relative Risks, we perform a meta-analysis for these studies. We also perform subgroup analyses in a sense that qualitatively, per category of determinants, we review if some determinants, compared to other determinants, are more often based on non-empirical reasoning rather than on empirical data and if allowed by the distribution of the data, also if they differ by study design or academic discipline (Box 2). We do not plan subgroup analyses for different forms of selective reporting because we anticipate that the exact outcome (e.g. selective reporting more generally or the bias that stems from it) is not always very clear in non-empirical publications.

**Meta bias(es)**

The proportion of articles that (quantitatively or qualitatively) report a non-significant or no association between a determinant and selective reporting is calculated, as we expect that non-significant findings are being reported as well. We also calculate the proportion of determinants for which at least once no association was found.

**Confidence in cumulative estimate**

The Grading of Recommendations Assessment, Development and Evaluation (GRADE approach) does not apply to our review. However, we summarize the confidence in the evidence in terms of types of studies and risk of bias these generally involve as explained under “Risk of bias of individual studies.”

**Discussion**

Using principles of both the systematic review and a qualitative content analysis, we aim to increase understanding of the nature of possible determinants of selective reporting. The search allows for identifying a broad base of possible determinants of selective reporting through not limiting to determinants that are identified a priori, in keeping with principles of qualitative research. The comprehensive analyses also cover both empirical findings and theoretical considerations. The search strategy focused on the outcome of selective reporting, regardless of, for example, design and also covers expert views. We extensively piloted possible search strategies and data extraction. We found that an efficient strategy included the use of a random sample of hits in a broad, sensitive search. This relates to the interpretation and discussion of a thorough qualitative content analyses of articles taking much time and skill.

**Limitations**

To restrict the review to a random sample means we may miss rarely mentioned or rarely studied determinants. This may be a concern if we find a substantial number of determinants only once. Further, by allowing the inclusion of all possible designs, and opinions obtained, for example, from discussion sections, we will be unable to consistently rate the quality of the publications. We also accept some duplication by including editorials. That is, it is possible that we include both an empirical article and an editorial highlighting the same study. We feel this is appropriate as most editorials also draw on the broader experience of the author.

**Implications**

The review identifies priority questions for further research on mechanisms of selective reporting. Determinants may represent causes, but also possible causes such as correlates, reported reasons or risk factors of selective reporting. This will help evidence-based development of interventions which is important because a recent review found that no empirical studies of current interventions such as prospective trial registries, have shown that they reduce publication bias [39]. Another recent review found that an important reason that authors report for not publishing conference reports is “lack of time” [40]. However, the quantitative and open-ended formats used do not permit for a detailed understanding of priorities or a balance between effort and expected gains.

Further, the review will help in assessing risk for selective reporting. “Risk profiling” may inform effective policy development on responsible conduct of research. For example, some research domains may receive little commercial funding, but sparse funding may increase the pressure to demonstrate positive output, and such a research domain may benefit from well-targeted interventions to prevent selective reporting.

**Dissemination**

We anticipate to use the review also for educational purposes, because of a determinant of selective reporting may be non-familiarity with the phenomenon, its pervasiveness and impact. An elaborate introduction to selective reporting is provided by a narrative (unsystematic) review of the state of knowledge. For this purpose, we will add an overview of what is known about selective reporting, including a taxonomy, estimates of its frequency of occurrence, and methods to assess it.

We will report the results, preferably in the shape of a peer-reviewed journal article with a broad readership. An abstract that announces the review has been presented at the 4th World Conference on Research Integrity Rio de Janeiro, May 31- June 3, 2015. The spreadsheets listing reviewed abstracts and the items abstracted from included articles will be made available, either upon request or directly accessible as a digital supplement.

Declarations

**Competing Interests**

None of the authors have any competing interests in the manuscript.

**Authors’ contributions**

JTS and LMB are the guarantors of the review. The study was conceived by JTS and LMB. All authors contributed to the development of the selection criteria. RHO, JTS and MCSP developed the search strategy. JTS allocated funding and drafted the manuscript. All authors read, provided feedback and approved the final manuscript.

**Acknowledgements**

**Funding**

The design of the study and writing of the article is funded by a career award for JTS from the Netherlands Organisation for Scientific Research (NWO; Innovational Research Incentives Scheme: Vidi grant number 917.11.339)

NWO provides salary support for JTS and MCSP and material support. The National Institute of Public Health and the Environment on behalf of the Dutch Ministry of Health, Welfare and Sports provide salary support for CAB. ZonMw the Netherlands Organisation for Health Research and Development, and the Academic Medical Center, University of Amsterdam, and Queen Mary University London provide salary support for GR. The salaries of the authors are provided by the VU University Medical Center (JTS, MCSP), Utrecht Institute for Pharmaceutical Sciences (UIPS) of Utrecht University (CAB), the University of Amsterdam (GR) and the VU University (RHJO, LMB). The funder, sponsors, and institutions had no role in the developing of the protocol.

**References**

1. Moorthy VS, Karam G, Vannice KS, Kieny MP. Rationale for WHO's New Position Calling for Prompt Reporting and Public Disclosure of Interventional Clinical Trial Results. PLoS Med. 2015;12(4):e1001819.

2. Chalmers I, Glasziou P. Avoiding waste in the production and reporting of research evidence. Lancet. 2009;374:86-9.

3. Jones CW, Handler L, Crowell KE, Keil LG, Weaver MA, Platts-Mills TF. Non-publication of large randomized clinical trials: cross sectional analysis. BMJ. 2013;347:f6104.

4. Ross JS, Mulvey GK, Hines EM, Nissen SE, Krumholz HM. Trial publication after registration in ClinicalTrials.Gov: a cross-sectional analysis. PLoS Med. 2009;6(9):e1000144.

5. Song F, Parekh S, Hooper L, Loke YK, Ryder J, Sutton AJ , et al. Dissemination and publication of research findings: an updated review of related biases. Health Technol Assess. 2010;14(8):iii,ix-xi,1-193.

6. Decullier E, Lhéritier V, Chapuis F. Fate of biomedical research protocols and publication bias in France: retrospective cohort study. BMJ. 2005;331(7507):19.

7. Petticrew M, Egan M, Thomson H, Hamilton V, Kunkler R, Roberts H. Publication bias in qualitative research: what becomes of qualitative research presented at conferences? J Epidemiol Community Health. 2008;62:552-4.

8. Hopewell S, Loudon K, Clarke MJ, Oxman AD, Dickersin K. Publication bias in clinical trials due to statistical significance or direction of trial results. Cochrane Database Syst Rev. 2009;(1):MR000006.

9. Cooper C, DeNeve K, Charlton K. Finding the missing science: the fate of studies submitted for review by a human subjects committee. Psychological Methods. 1997;2(4):447-52.

10. Song F, Eastwood AJ, Gilbody S, Duley L, Sutton AJ. Publication and related biases. Health Technol Assess. 2000;4(10):1-115.

11. Jones CW, Handler L, Crowell KE, Keil LG, Weaver MA, Platts-Mills TF. Non-publication of large randomized clinical trials: cross sectional analysis. BMJ. 2013;347:f6104.

12. McGauran N, Wieseler B, Kreis J, Schüler YB, Kölsch H, Kaiser T. Reporting bias in medical research - a narrative review. Trials. 2010;11:37.

13. Greenland S. Accounting for uncertainty about investigator bias: disclosure is informative. J Epidemiol Community Health. 2009;63(8):593-8.

14. Dunkelberg S. Further information on publication bias. [Weiterführende Informationen zu Publication bias]. Z Allg Med. 2005;81:145–6.

15. Martinson BC, Anderson MS, Crain AL, de Vries R. Scientists' perceptions of organizational justice and self-reported misbehaviors. J Empir Res Hum Res Ethics. 2006;1(1):51-66.

16. Fanelli D. Do pressures to publish increase scientists' bias? An empirical support from US States data. PLoS One. 2010;5(4):e10271.

17. Moher D, Shamseer L, Clarke M, Ghersi D, Liberati A, Petticrew M et al*.*; PRISMA-P Group. Preferred reporting items for systematic review and meta-analysis protocols (PRISMA-P) 2015 statement. *Syst Rev.* 2015;4(1):1.

18. Shamseer L, Moher D, Clarke M, Ghersi D, Liberati A, Petticrew M et al.; PRISMA-P Group. Preferred reporting items for systematic review and meta-analysis protocols (PRISMA-P) 2015: elaboration and explanation. *BMJ.* 2015;349:g7647.

19. Smyth RM, Kirkham JJ, Jacoby A, Altman DG, Gamble C, Williamson PR. Frequency and reasons for outcome reporting bias in clinical trials: interviews with trialists. BMJ. 2011;342:c7153.

20. Djulbegovic B, Kumar A, Magazin A, Schroen AT, Soares H, Hozo I, et al. Optimism bias leads to inconclusive results-an empirical study. J Clin Epidemiol. 2011;64:583-93.

21. Chalmers I, Glasziou P, Godlee F. All trials must be registered and the results published. BMJ. 2013;346:f105.

22. Dwan K, Altman DG, Cresswell L, Blundell M, Gamble CL, Williamson PR. Comparison of protocols and registry entries to published reports for randomised controlled trials. Cochrane Database Syst Rev. 2011;(1):MR000031.

23. Cuijpers P, Smit F, Bohlmeijer E, Hollon SD, Andersson G. Efficacy of cognitive-behavioural therapy and other psychological treatments for adult depression: meta-analytic study of publication bias. Br J Psychiatry. 2010;196:173-8.

24. Okike K, Kocher MS, Nwachukwu BU, Mehlman CT, Heckman JD, Bhandari M. The fate of manuscripts rejected by The Journal of Bone and Joint Surgery (American Volume). J Bone Joint Surg Am. 2012;94(17):e130.

25. Dickersin K, Chalmers I. Recognizing, investigating and dealing with incomplete and biased reporting of clinical research: from Francis Bacon to the WHO. J R Soc Med. 2011;104:532-8.

26. O’Boyle EH, Banks GC, Gonzalez-Mulé E. The Chrystalis effect: how ugly initial result metamorphosize into beautiful articles. Journal of Management. 2014, March 19: 0149206314527133.

27. Shakiba B, Salmasian H, Yousefi-Nooraie R, Rohanizadegan M. Factors influencing editors' decision on acceptance or rejection of manuscripts: the authors' perspective. Arch Iran Med. 2008;11:257-62.

28. Ware JJ, Munafò MR. Significance chasing in research practice: causes, consequences and possible solutions. Addiction. 2015;110(1):4-8.

29. Ioannidis JP, Munafò MR, Fusar-Poli P, Nosek BA, David SP. Publication and other reporting biases in cognitive sciences: detection, prevalence, and prevention. Trends Cogn Sci .2014;18:235-41.

30. Ioannidis JP. How to make more published research true. PLoS Med. 2014;11(10):e1001747.

31. Fanelli D. "Positive" results increase down the Hierarchy of the Sciences. PLoS One. 2010;5(4):e10068.

32. Ioannidis JP. Why most published research findings are false. PLoS Med. 2005;2(8):e124.

33. Chalmers TC, Frank CS, Reitman D. Minimizing the three stages of publication bias. JAMA. 1990;263:1392-5.

34. Cochrane Handbook for Systematic Reviews of Interventions Version 5.1.0: updated March 2011. Edited by Higgins JPT, Green S. The Cochrane Collaboration; 2011 [www.cochrane-handbook.org]

35. Olsen KL, Hopewell S, Dickersin K, Clarke M, Oxman AD. Publication bias in clinical trials. Editorial Group: Cochrane Methodology Review Group. Methodology Protocol. Published Online: 23 JUL 2001. DOI: 10.1002/14651858.MR000006; Update published Online: 21 JAN 2009. DOI: 10.1002/14651858.MR000006.pub3.

36. Pace R, Pluye P, Bartlett G, Macaulay AC, Salsberg J, Jagosh J, Seller R. Testing the reliability and efficiency of the pilot Mixed Methods Appraisal Tool (MMAT) for systematic mixed studies review. Int J Nurs Stud. 2012;49(1):47-53.

37. van der Steen JT, van Soest-Poortvliet MC, Hallie-Heierman M, Onwuteaka-Philipsen BD, Deliens L, de Boer ME, et al. Factors associated with initiation of advance care planning in dementia: a systematic review. J Alzheimers Dis. 2014;40:743-57.

38. Vaismoradi M, Turunen H, Bondas T. Content analysis and thematic analysis: Implications for conducting a qualitative descriptive study. Nurs Health Sci. 2013;15:398-405.

39. Thaler K, Kien C, Nussbaumer B, Van Noord MG, Griebler U, Klerings I, Gartlehner G; UNCOVER Project Consortium. Inadequate use and regulation of interventions against publication bias decreases their effectiveness: a systematic review. J Clin Epidemiol. 2015;68(7):792-802.

40. Scherer RW, Ugarte-Gil C, Schmucker C, Meerpohl JJ. Authors report lack of time as main reason for unpublished research presented at biomedical conferences: a systematic review. J Clin Epidemiol. 2015l;68(7):803-10.

Selective Reporting

Non-Publication Selective (reporting within) Publication

← focus on **preferred** (often “positive”) findings →

Publication Bias e.g. Outcome Reporting Bias

Reporting Bias

**Figure 1** **The review’s outcome selective reporting, forms and resulting bias**

**Box 1** **Search strategy**

| **Search strategy as defined in the PubMed database**  1. "Publication Bias"[Majr]  2. "Publishing"[Majr] AND "Bias (Epidemiology)"[Majr]  3. #1 OR #2  4. reporting bias*[ti] OR biased reporting[ti] OR selective reporting[ti] OR selective publication*[ti] OR publication bias*[ti] OR nonpublication*[ti] OR non-publication*[ti]  5. #3 OR #4  6. Search #5 AND ("dutch"[Language] OR "french"[Language] OR "english"[Language] OR "german"[Language])  7. Search #6 NOT ("addresses"[Publication Type] OR "biography"[Publication Type] OR "comment"[Publication Type] OR "directory"[Publication Type] OR "festschrift"[Publication Type] OR "interview"[Publication Type] OR "lectures"[Publication Type] OR "legal cases"[Publication Type] OR "legislation"[Publication Type] OR "letter"[Publication Type] OR "news"[Publication Type] OR "newspaper article"[Publication Type] OR "patient education handout"[Publication Type] OR "popular works"[Publication Type] OR "congresses"[Publication Type])  **Summary of the congruent search strategies in the other databases**  We also searched Embase.com, PsycINFO (via EBSCO) and Web of Science. We used the MeSH terms as title word (reporting bias, etc.), except for Embase where we used also ‘publishing' AND 'statistical bias' as major Emtree terms. |
| --- |

**Box 2** **Items abstracted from included articles**

| Item | Response options |
| --- | --- |
| a. Academic discipline  (origin or topic) | -(clinical) medicine  -biomedicine, life sciences, technology  -humanities (e.g. history, language)  -sciences (beta; natural, formal);  -society and behaviour (gamma, e.g. psychology, economics)  -other  -mixed |
| b. Study design | -theoretical consideration/reflection (includes view beyond direct interpretation of empirical study results)  -case report/anecdote  -qualitative research  -quantitative observational longitudinal  -quantitative observational cross-sectional  -non-randomized intervention (quasi-experimental, CCT)  -randomized intervention  -mixed methods (integrated)  -any other design |
| *The items below are all reported at the level of the determinant* | |
| c. Description of the determinant | Open-ended text item. *More determinants possible*.  Also copy phrases that include how the determinant is described or interpreted in the manuscript, e.g. cause, risk factor, reason. |
| d. Level of evidence for determinant (section) | -view (introduction, discussion, or opinion piece)  -empirical data |
| e. Measurement  (for quantitative studies only) | -objective measurement of determinant and outcome  -any subjective measurement (e.g. self report) |
| f. Type of study  (for observational studies only) | -comparison of registration (ethics or regulatory) with publication  -comparison of protocol with publication  -comparison of abstract with publication  -other, e.g. survey |
| g. Type of outcome | -non-publication  -selective reporting within publication  -selective reporting more generally  -reporting bias  -other (might include a focus on preferred findings as an outcome)  -not specified |
| h. Scope of outcome | -reporting generally  -reporting within a specific medium, e.g. a journal |
| i. Reported / found an association | -yes  -no (e.g. not significant)  -unclear |
| j. Specify direction of the association  (if any) | Open-ended text item |
| k. Strength of the association | -report measure (e.g. effect size) for quantitative association, if (un)adjusted, and precision (confidence interval, or significance)  -also report qualitative relationships (e.g. most or more important cause) |
| *The items below are reported at the level of the article* | |
| l. Preventive measures, remedies | Open-ended text item. For example, registration of study protocol and early data analysis plan (more possible). |
| m. Any comments | Open-ended text item |
