# Supplementary material for: Determinants of selective reporting: A taxonomy based on content analysis of a random selection of the literature
Source: PLoS One. 2018 Feb 5;13(2):e0188247. doi: 10.1371/journal.pone.0188247 (PMC5798766; doi:10.1371/journal.pone.0188247)
Supplement: S3 File — (DOCX) [file pone.0188247.s004.docx]

S3 Supplemental file

**Table. References to the 64 articles included in the determinant analysis, per category**

| Determinant classification, category | Citations of articles from which determinant was extracted^*^ |
| --- | --- |
| *1. Focus on preferred findings* | [[1-52](#_ENREF_1)] |
| *2. Poor or flexible research design* | [[1](#_ENREF_1), [4](#_ENREF_4), [6](#_ENREF_6), [7](#_ENREF_7), [9](#_ENREF_9), [10](#_ENREF_10), [12-19](#_ENREF_12), [21-25](#_ENREF_21), [27-32](#_ENREF_27), [38](#_ENREF_38), [40](#_ENREF_40), [41](#_ENREF_41), [44](#_ENREF_44), [48](#_ENREF_48), [49](#_ENREF_49), [53-55](#_ENREF_53)] |
| *3. High-risk area and its development* | [[3-5](#_ENREF_3), [7](#_ENREF_7), [9-12](#_ENREF_9), [19](#_ENREF_19), [21](#_ENREF_21), [23](#_ENREF_23), [25](#_ENREF_25), [27](#_ENREF_27), [29](#_ENREF_29), [30](#_ENREF_30), [32](#_ENREF_32), [41](#_ENREF_41), [45](#_ENREF_45), [47](#_ENREF_47), [49](#_ENREF_49), [53-59](#_ENREF_53)] |
| *4. Dependence upon sponsors* | [[1](#_ENREF_1), [3](#_ENREF_3), [4](#_ENREF_4), [13](#_ENREF_13), [16](#_ENREF_16), [19](#_ENREF_19), [21](#_ENREF_21), [22](#_ENREF_22), [24](#_ENREF_24), [25](#_ENREF_25), [27](#_ENREF_27), [31](#_ENREF_31), [33](#_ENREF_33), [36](#_ENREF_36), [38](#_ENREF_38), [40](#_ENREF_40), [49-51](#_ENREF_49), [60](#_ENREF_60)] |
| *5. Prejudice* | [[3](#_ENREF_3), [8](#_ENREF_8), [19](#_ENREF_19), [22-24](#_ENREF_22), [27](#_ENREF_27), [29](#_ENREF_29), [31](#_ENREF_31), [32](#_ENREF_32), [40](#_ENREF_40), [41](#_ENREF_41), [45](#_ENREF_45), [49](#_ENREF_49), [53](#_ENREF_53), [61](#_ENREF_61), [62](#_ENREF_62)] |
| *6. Lack of resources including time* | [[1](#_ENREF_1), [19](#_ENREF_19), [21](#_ENREF_21), [23](#_ENREF_23), [36](#_ENREF_36), [45](#_ENREF_45), [50](#_ENREF_50), [53](#_ENREF_53), [55](#_ENREF_55), [59](#_ENREF_59), [63](#_ENREF_63)] |
| *7. Doubts about reporting being worth the effort* | [[1](#_ENREF_1), [16](#_ENREF_16), [30](#_ENREF_30), [45](#_ENREF_45), [47](#_ENREF_47), [49](#_ENREF_49), [50](#_ENREF_50), [59](#_ENREF_59)] |
| *8. Limitations in reporting and editorial practices* | [[2](#_ENREF_2), [7](#_ENREF_7), [10](#_ENREF_10), [21](#_ENREF_21), [23](#_ENREF_23), [33](#_ENREF_33)] |
| *9. Academic publication system hurdles* | [[7](#_ENREF_7), [9](#_ENREF_9), [23](#_ENREF_23), [25](#_ENREF_25), [36](#_ENREF_36), [47](#_ENREF_47), [49](#_ENREF_49), [53](#_ENREF_53)] |
| *10. Unfavorable geographical or regulatory environment^§^* | [[1](#_ENREF_1), [3](#_ENREF_3), [9](#_ENREF_9), [10](#_ENREF_10), [19](#_ENREF_19), [25](#_ENREF_25), [31](#_ENREF_31), [38](#_ENREF_38), [60](#_ENREF_60)] |
| *11. Relationship and collaboration issues* | [[4](#_ENREF_4), [24](#_ENREF_24), [34](#_ENREF_34), [38](#_ENREF_38), [45](#_ENREF_45), [63](#_ENREF_63)] |
| *12. Potential harm* | [[6](#_ENREF_6), [64](#_ENREF_64)] |
| (13) Not specified | [[13](#_ENREF_13), [19](#_ENREF_19), [22](#_ENREF_22), [34](#_ENREF_34), [45](#_ENREF_45), [63](#_ENREF_63)] |

**References**

1. Chapman SJ, Shelton B, Mahmood H, Fitzgerald JE, Harrison EM, Bhangu A. Discontinuation and non-publication of surgical randomised controlled trials: observational study. BMJ. 2014;349:g6870. Epub 2014/12/11. doi: 10.1136/bmj.g6870. PubMed PMID: 25491195; PubMed Central PMCID: PMC4260649.

2. Dal-Re R, Caplan AL. Time to ensure that clinical trial appropriate results are actually published. Eur J Clin Pharmacol. 2014;70(4):491-3. Epub 2014/01/15. doi: 10.1007/s00228-013-1635-0. PubMed PMID: 24413686.

3. Dieterich S, Keall PJ, Orton CG. Point/Counterpoint. Increasing dependence on industry-funded research creates higher risk of biased reporting in medical physics. Med Phys. 2013;40(10):100601. Epub 2013/10/05. doi: 10.1118/1.4812894. PubMed PMID: 24089887.

4. Ferguson CJ, Brannick MT. Publication bias in psychological science: prevalence, methods for identifying and controlling, and implications for the use of meta-analyses. Psychol Methods. 2012;17(1):120-8. Epub 2011/07/27. doi: 10.1037/a0024445. PubMed PMID: 21787082.

5. Mathieu S, Boutron I, Moher D, Altman DG, Ravaud P. Comparison of registered and published primary outcomes in randomized controlled trials. JAMA. 2009;302(9):977-84. Epub 2009/09/03. doi: 10.1001/jama.2009.1242. PubMed PMID: 19724045.

6. Mattila T, Stoyanova V, Elferink A, Gispen-de Wied C, de Boer A, Wohlfarth T. Insomnia medication: do published studies reflect the complete picture of efficacy and safety? Eur Neuropsychopharmacol. 2011;21(7):500-7. Epub 2010/11/19. doi: 10.1016/j.euroneuro.2010.10.005. PubMed PMID: 21084176.

7. Onishi A, Furukawa TA. Publication bias is underreported in systematic reviews published in high-impact-factor journals: metaepidemiologic study. J Clin Epidemiol. 2014;67(12):1320-6. Epub 2014/09/10. doi: 10.1016/j.jclinepi.2014.07.002. PubMed PMID: 25194857.

8. Park IU, Peacey MW, Munafo MR. Modelling the effects of subjective and objective decision making in scientific peer review. Nature. 2014;506(7486):93-6. Epub 2013/12/07. doi: 10.1038/nature12786. PubMed PMID: 24305052.

9. Salami K, Alkayed K. Publication bias in pediatric hematology and oncology: analysis of abstracts presented at the annual meeting of the American Society of Pediatric Hematology and Oncology. Pediatr Hematol Oncol. 2013;30(3):165-9. Epub 2013/03/07. doi: 10.3109/08880018.2013.774078. PubMed PMID: 23461713.

10. Sando IC, Malay S, Chung KC. Analysis of publication bias in the literature for distal radius fracture. J Hand Surg Am. 2013;38(5):927-34 e5. Epub 2013/04/10. doi: 10.1016/j.jhsa.2013.02.023. PubMed PMID: 23566720; PubMed Central PMCID: PMC3667615.

11. Simundic AM. Bias in research. Biochem Med (Zagreb). 2013;23(1):12-5. Epub 2013/03/06. PubMed PMID: 23457761; PubMed Central PMCID: PMC3900086.

12. Smulders YM. A two-step manuscript submission process can reduce publication bias. J Clin Epidemiol. 2013;66(9):946-7. Epub 2013/07/13. doi: 10.1016/j.jclinepi.2013.03.023. PubMed PMID: 23845183.

13. Sune P, Sune JM, Montoro JB. Positive outcomes influence the rate and time to publication, but not the impact factor of publications of clinical trial results. PLoS One. 2013;8(1):e54583. Epub 2013/02/06. doi: 10.1371/journal.pone.0054583. PubMed PMID: 23382919; PubMed Central PMCID: PMC3559840.

14. Trinquart L, Ioannidis JP, Chatellier G, Ravaud P. A test for reporting bias in trial networks: simulation and case studies. BMC Med Res Methodol. 2014;14:112. Epub 2014/09/30. doi: 10.1186/1471-2288-14-112. PubMed PMID: 25262204; PubMed Central PMCID: PMC4193287.

15. Tzoulaki I, Siontis KC, Evangelou E, Ioannidis JP. Bias in associations of emerging biomarkers with cardiovascular disease. JAMA Intern Med. 2013;173(8):664-71. Epub 2013/03/27. doi: 10.1001/jamainternmed.2013.3018. PubMed PMID: 23529078.

16. Abaid LN, Grimes DA, Schulz KF. Reducing publication bias through trial registration. Obstet Gynecol. 2007;109(6):1434-7. Epub 2007/06/02. doi: 10.1097/01.AOG.0000266557.11064.2a. PubMed PMID: 17540818.

17. Anderson HR, Atkinson RW, Peacock JL, Sweeting MJ, Marston L. Ambient particulate matter and health effects: publication bias in studies of short-term associations. Epidemiology. 2005;16(2):155-63. Epub 2005/02/11. PubMed PMID: 15703529.

18. Bardy AH. Bias in reporting clinical trials. Br J Clin Pharmacol. 1998;46(2):147-50. Epub 1998/09/02. PubMed PMID: 9723823; PubMed Central PMCID: PMC1873669.

19. Begg CB, Berlin JA. Publication bias and dissemination of clinical research. J Natl Cancer Inst. 1989;81(2):107-15. Epub 1989/01/18. PubMed PMID: 2642556.

20. Chalmers I. From optimism to disillusion about commitment to transparency in the medico-industrial complex. J R Soc Med. 2006;99(7):337-41. Epub 2006/07/04. doi: 10.1258/jrsm.99.7.337. PubMed PMID: 16816262; PubMed Central PMCID: PMC1484602.

21. Chan AW, Altman DG. Identifying outcome reporting bias in randomised trials on PubMed: review of publications and survey of authors. BMJ. 2005;330(7494):753. Epub 2005/02/01. doi: 10.1136/bmj.38356.424606.8F. PubMed PMID: 15681569; PubMed Central PMCID: PMC555875.

22. Dickersin K, Min YI. NIH clinical trials and publication bias. Online J Curr Clin Trials. 1993;Doc No 50:[4967 words; 53 paragraphs]. Epub 1993/04/28. PubMed PMID: 8306005.

23. Gilbert JR, Williams ES, Lundberg GD. Is there gender bias in JAMA's peer review process? JAMA. 1994;272(2):139-42. Epub 1994/07/13. PubMed PMID: 8015126.

24. Greenland S. Accounting for uncertainty about investigator bias: disclosure is informative. J Epidemiol Community Health. 2009;63(8):593-8. Epub 2009/07/15. doi: 10.1136/jech.2008.084913. PubMed PMID: 19596837.

25. Harris IA, Mourad M, Kadir A, Solomon MJ, Young JM. Publication bias in abstracts presented to the annual meeting of the American Academy of Orthopaedic Surgeons. J Orthop Surg (Hong Kong). 2007;15(1):62-6. Epub 2007/04/13. PubMed PMID: 17429120.

26. Hopewell S, Clarke M, Stewart L, Tierney J. Time to publication for results of clinical trials. Cochrane Database Syst Rev. 2007;(2):MR000011. Epub 2007/04/20. doi: 10.1002/14651858.MR000011.pub2. PubMed PMID: 17443632.

27. Ioannidis JP. Why most published research findings are false. PLoS Med. 2005;2(8):e124. Epub 2005/08/03. doi: 10.1371/journal.pmed.0020124. PubMed PMID: 16060722; PubMed Central PMCID: PMC1182327.

28. Nassir Ghaemi S, Shirzadi AA, Filkowski M. Publication bias and the pharmaceutical industry: the case of lamotrigine in bipolar disorder. Medscape J Med. 2008;10(9):211. Epub 2008/11/15. PubMed PMID: 19008973; PubMed Central PMCID: PMC2580079.

29. Newcombe RG. Towards a reduction in publication bias. Br Med J (Clin Res Ed). 1987;295(6599):656-9. Epub 1987/09/12. PubMed PMID: 3117278; PubMed Central PMCID: PMC1257777.

30. Okike K, Kocher MS, Mehlman CT, Heckman JD, Bhandari M. Publication bias in orthopaedic research: an analysis of scientific factors associated with publication in the Journal of Bone and Joint Surgery (American Volume). J Bone Joint Surg Am. 2008;90(3):595-601. Epub 2008/03/04. doi: 10.2106/JBJS.G.00279. PubMed PMID: 18310710.

31. Olson CM, Rennie D, Cook D, Dickersin K, Flanagin A, Hogan JW, et al. Publication bias in editorial decision making. JAMA. 2002;287(21):2825-8. Epub 2002/06/01. PubMed PMID: 12038924.

32. Preston C, Ashby D, Smyth R. Adjusting for publication bias: modelling the selection process. J Eval Clin Pract. 2004;10(2):313-22. Epub 2004/06/11. doi: 10.1111/j.1365-2753.2003.00457.x. PubMed PMID: 15189397.

33. Rampton S, Stauber J. Research funding, conflicts of interest, and the meta-methodology of public relations. Public Health Rep. 2002;117(4):331-9. Epub 2002/12/13. PubMed PMID: 12477914; PubMed Central PMCID: PMC1497451.

34. Rifai N, Altman DG, Bossuyt PM. Reporting bias in diagnostic and prognostic studies: time for action. Clin Chem. 2008;54(7):1101-3. Epub 2008/07/03. doi: 10.1373/clinchem.2008.108993. PubMed PMID: 18593957.

35. Steinbrook R. Public registration of clinical trials. N Engl J Med. 2004;351(4):315-7. Epub 2004/07/23. doi: 10.1056/NEJMp048191. PubMed PMID: 15269307.

36. Stewart LA, Parmar MK. Bias in the analysis and reporting of randomized controlled trials. Int J Technol Assess Health Care. 1996;12(2):264-75. Epub 1996/01/01. PubMed PMID: 8707499.

37. Takeda A, Loveman E, Harris P, Hartwell D, Welch K. Time to full publication of studies of anti-cancer medicines for breast cancer and the potential for publication bias: a short systematic review. Health Technol Assess. 2008;12(32):iii, ix-x, 1-46. Epub 2008/10/04. PubMed PMID: 18831948.

38. von Elm E, Rollin A, Blumle A, Huwiler K, Witschi M, Egger M. Publication and non-publication of clinical trials: longitudinal study of applications submitted to a research ethics committee. Swiss Med Wkly. 2008;138(13-14):197-203. Epub 2008/04/05. doi: 2008/13/smw-12027. PubMed PMID: 18389392.

39. The time is coming. Nat Rev Drug Discov. 2004;3(11):897-8. Epub 2004/11/24. doi: 10.1038/nrd1567. PubMed PMID: 15558855.

40. de Melker HE, Rosendaal FR, Vandenbroucke JP. [The importance of publication bias in medical-scientific literature]. Ned Tijdschr Geneeskd. 1993;137(42):2126-30. Epub 1993/10/16. PubMed PMID: 8232658.

41. Ferguson CJ, Heene M. A Vast Graveyard of Undead Theories: Publication Bias and Psychological Science's Aversion to the Null. Perspect Psychol Sci. 2012;7(6):555-61. Epub 2012/11/01. doi: 10.1177/1745691612459059. PubMed PMID: 26168112.

42. Guemann M. Registration of clinical trials in physiotherapy, an ISPJE's recommendation to fight against the publication biases. Kinesitherapie. 2013;13(137):1.

43. Hoye A, Elvik R. Publication Bias in Road Safety Evaluation How Can It Be Detected and How Common Is It? Transp Res Record. 2010;(2147):1-8. doi: 10.3141/2147-01. PubMed PMID: WOS:000284180600001.

44. Lang L. New Study Finds Publication Bias Among Trials Submitted to the US Food and Drug Administration. Gastroenterology. 2009;136(2):374.

45. McCambridge J. A response to the commentaries: look away now or face up to the profound problem of publication bias in drug education research. Drug Alcohol Rev. 2008;27(4):352-6. doi: 10.1080/09595230802090097. PubMed PMID: WOS:000257147500007.

46. McDaniel MA, Rothstein HR, Whetzel DL. Publication bias: A case study of four test vendors. Pers Psychol. 2006;59(4):927-53. doi: 10.1111/j.1744-6570.2006.00059.x. PubMed PMID: WOS:000242534800006.

47. Parmley WW. Publication bias. J Am Coll Cardiol. 1994;24(5):1424-5. Epub 1994/11/01. PubMed PMID: 7930269.

48. Rothstein HR, Bushman BJ. Publication bias in psychological science: comment on Ferguson and Brannick (2012). Psychol Methods. 2012;17(1):129-36. Epub 2012/03/01. doi: 10.1037/a0027128. PubMed PMID: 22369520.

49. Shields PG. Publication bias is a scientific problem with adverse ethical outcomes: the case for a section for null results. Cancer Epidemiol Biomarkers Prev. 2000;9(8):771-2. Epub 2000/08/22. PubMed PMID: 10952092.

50. Song F, Hooper L, Loke YK. Publication bias: What is it? How do we measure it? How do we avoid it? Reports in Medical Imaging. 2012;5(1):71-81.

51. Bardy AH. Report bias in drug research. Therapie. 1996;51(4):382-3. Epub 1996/07/01. PubMed PMID: 8953813.

52. Reidenberg MM. Decreasing publication bias. Clin Pharmacol Ther. 1998;63(1):1-3. Epub 1998/02/18. doi: 10.1016/S0009-9236(98)90115-1. PubMed PMID: 9465836.

53. Goldsmith LA, Blalock EN, Bobkova H, Hall RP, 3rd. Picking your peers. J Invest Dermatol. 2006;126(7):1429-30. Epub 2006/06/17. doi: 10.1038/sj.jid.5700387. PubMed PMID: 16778804.

54. Moher D, Fortin P, Jadad AR, Juni P, Klassen T, Le Lorier J, et al. Completeness of reporting of trials published in languages other than English: implications for conduct and reporting of systematic reviews. Lancet. 1996;347(8998):363-6. Epub 1996/02/10. PubMed PMID: 8598702.

55. Negrini S. Another form of publication bias: the unpublished "everyday" clinical researches. Eur J Phys Rehabil Med. 2011;47(4):531-2. Epub 2012/01/10. PubMed PMID: 22222957.

56. Altman DG. Poor-quality medical research: what can journals do? JAMA. 2002;287(21):2765-7. Epub 2002/06/01. PubMed PMID: 12038906.

57. Hopewell S, McDonald S. Full publication of trials initially reported as abstracts in the Australian and New Zealand Journal of Medicine 1980-2000. Intern Med J. 2003;33(4):192-4. Epub 2003/04/12. PubMed PMID: 12680987.

58. Munafo MR, Flint J. How reliable are scientific studies? Br J Psychiatry. 2010;197(4):257-8. Epub 2010/10/05. doi: 10.1192/bjp.bp.109.069849. PubMed PMID: 20884944.

59. Sherrod J. Selective publication of information. Spec Libr. 1964;55(6):386-7. PubMed PMID: WOS:A1964CEH3000010.

60. Bourgeois FT, Murthy S, Mandl KD. Outcome reporting among drug trials registered in ClinicalTrials.gov. Ann Intern Med. 2010;153(3):158-66. Epub 2010/08/04. doi: 10.7326/0003-4819-153-3-201008030-00006. PubMed PMID: 20679560; PubMed Central PMCID: PMC3374868.

61. Fisher M, Friedman SB, Strauss B. The effects of blinding on acceptance of research papers by peer review. JAMA. 1994;272(2):143-6. Epub 1994/07/13. PubMed PMID: 8015127.

62. Pless B. Are Editors free from bias? The special case of Letters to the Editor. Inj Prev. 2006;12(6):353-4. Epub 2006/12/16. doi: 10.1136/ip.2006.014472. PubMed PMID: 17170177; PubMed Central PMCID: PMC2564407.

63. Dowd MD. Breaching the contract: the ethics of nonpublication of research studies. Arch Pediatr Adolesc Med. 2004;158(10):1014-5. Epub 2004/10/07. doi: 10.1001/archpedi.158.10.1014. PubMed PMID: 15466692.

64. Gyles C. Is there ever good reason to not publish good science? Can Vet J. 2012;53(6):587-8. Epub 2012/12/04. PubMed PMID: 23204577; PubMed Central PMCID: PMC3354816.
